# Prevalence of pressure injuries in Japanese older people: A population-based cross-sectional study

**DOI:** 10.1371/journal.pone.0198073

**Published:** 2018-06-07

**Authors:** Shuji Nakashima, Hirotomo Yamanashi, Satomi Komiya, Katsumi Tanaka, Takahiro Maeda

**Affiliations:** 1 Nagasaki Prefecture Tomie Hospital, Goto, Nagasaki, Japan; 2 Department of Island and Community Medicine, Nagasaki University Graduate School of Biomedical Sciences, Goto, Nagasaki, Japan; 3 Department of Clinical Medicine, Institute of Tropical Medicine, Nagasaki University, Sakamoto, Nagasaki, Japan; 4 Nagasaki Prefecture Goto Central Hospital, Goto, Nagasaki, Japan; 5 Department of Plastic and Reconstructive Surgery, Nagasaki University Graduate School of Biomedical Sciences, Sakamoto, Nagasaki, Japan; 6 Department of Community Medicine, Nagasaki University Graduate School of Biomedical Sciences, Sakamoto, Nagasaki, Japan; University of Illinois at Urbana-Champaign, UNITED STATES

## Abstract

**Objectives:**

The prevalence of pressure injuries is an essential indicator of prevention and quality of care. Population-based prevalence data on pressure injuries are scarce in Japan. This study aimed to estimate the prevalence of pressure injuries per 1000 adults and per 1000 older people in Japan.

**Design:**

Cross-sectional survey.

**Setting:**

This study was conducted in Goto, a city located on a remote rural archipelago in Japan. In 2017, the population was 37,855; older people aged ≥65 years accounted for 37.7%.

**Participants:**

Participants were enrolled in various facilities in the city. In total, 1126 participants (median age 85 years) were assessed to calculate age-specific numbers of people with pressure injuries.

**Measurements:**

Participants were directly evaluated by the research team between August and September 2017, and pressure injuries were classified using DESIGN-R schema. We calculated the number of adults with pressure injuries in Goto based on the proportion of pressure injuries in specific age categories. In these prevalence estimations, we assumed that all cases aged ≥65 years were long-term care insurance-certified older people, and all cases aged 18–64 years were people with physical disabilities who received social welfare services.

**Results:**

Of the 1126 participants, 113 (10%) had one or more pressure injuries. Overall, the estimated number of adults with pressure injuries in Goto was 301.4. The prevalence rate of pressure injuries was 9.2 per 1000 population in adults aged ≥18 years (95% confidence interval [CI] 8.1–10.2), 20.3 in those aged ≥65 years (95% CI 18.1–22.7), and 44.6 in those aged ≥80 years (95% CI 39.5–50.2).

**Conclusions:**

This study revealed a high population-based prevalence of pressure injuries in a rural Japanese community. A key reason for this high disease burden in Japan appears to be the susceptibility of the aged population to pressure injuries.

## Introduction

Pressure injuries greatly affect health-related quality of life [1] and impose significant burdens, including affective discomfort from pain, unpleasant wound odours, social isolation, financial problems, emotional stress from being dependent on others and mortality [2–4]. Pressure injuries cost US$9.1–US$11.6 billion per year in the United States and £1.4– £2.1 billion per year in the United Kingdom; treating pressure injuries can become increasingly expensive depending on severity [5–7].

Age, immobility, inactivity, skin pressure, diabetes, vascular disease, skin moisture and haematological or nutritional status are known risk factors for pressure injuries [3,8,9]. A UK population-based study reported the aged population accounted for a high proportion of pressure injuries [10]. The number of community-dwelling older people susceptible to pressure injuries is continuing to rise. Prevention of pressure injuries and reducing severe cases in the community are therefore major public health concerns.

Several quality indicators have been proposed as qualified prevention and care for pressure injuries. These include risk assessment tools (e.g. Braden Scale), nutritional assessment, pressure injury evaluation and topical treatments [11]. Although these indicators are useful to assess the quality of care for pressure injuries at an individual or facility level, a comparable indicator is needed in public health.

The prevalence rate of pressure injuries can provide a useful community-level indicator to evaluate the success of prevention protocols and ensure fair application of financial incentive/penalty schemes [12]. However, there are several essential points in ensuring epidemiological studies produce accurate results that allow valid comparisons among facilities [12,13]. For example, the selection of facilities for data collection, method of identifying cases, and definition of the study population as the denominator are crucial determinants in prevalence studies [12]. Another important focus area is transitions of care in older people. Older people frequently require transitions of care between home, home with formal services, hospital and nursing facility care [14,15]. Consequently, facility-based prevalence study designs have strong selection bias because individual facilities have functional roles in their community that are influenced by governmental policy or bed capacity (e.g. available number of beds in hospitals, long-term care facilities and palliative care facilities). Therefore, we proposed a population-based study design that included different types of facilities in the targeted community to investigate the prevalence of pressure injuries.

Japan’s population is aging rapidly because of the long life expectancy and low birth rate, and investigation of the prevalence of pressure injuries is urgently needed. Although several facility-based prevalence studies have been conducted, no population-based studies have been conducted to date in Japan [16–18]. This study aimed to investigate the population-based prevalence of pressure injuries in Japanese adults and older adults.

## Materials and methods

### Study setting and participants

**Fig 1:**
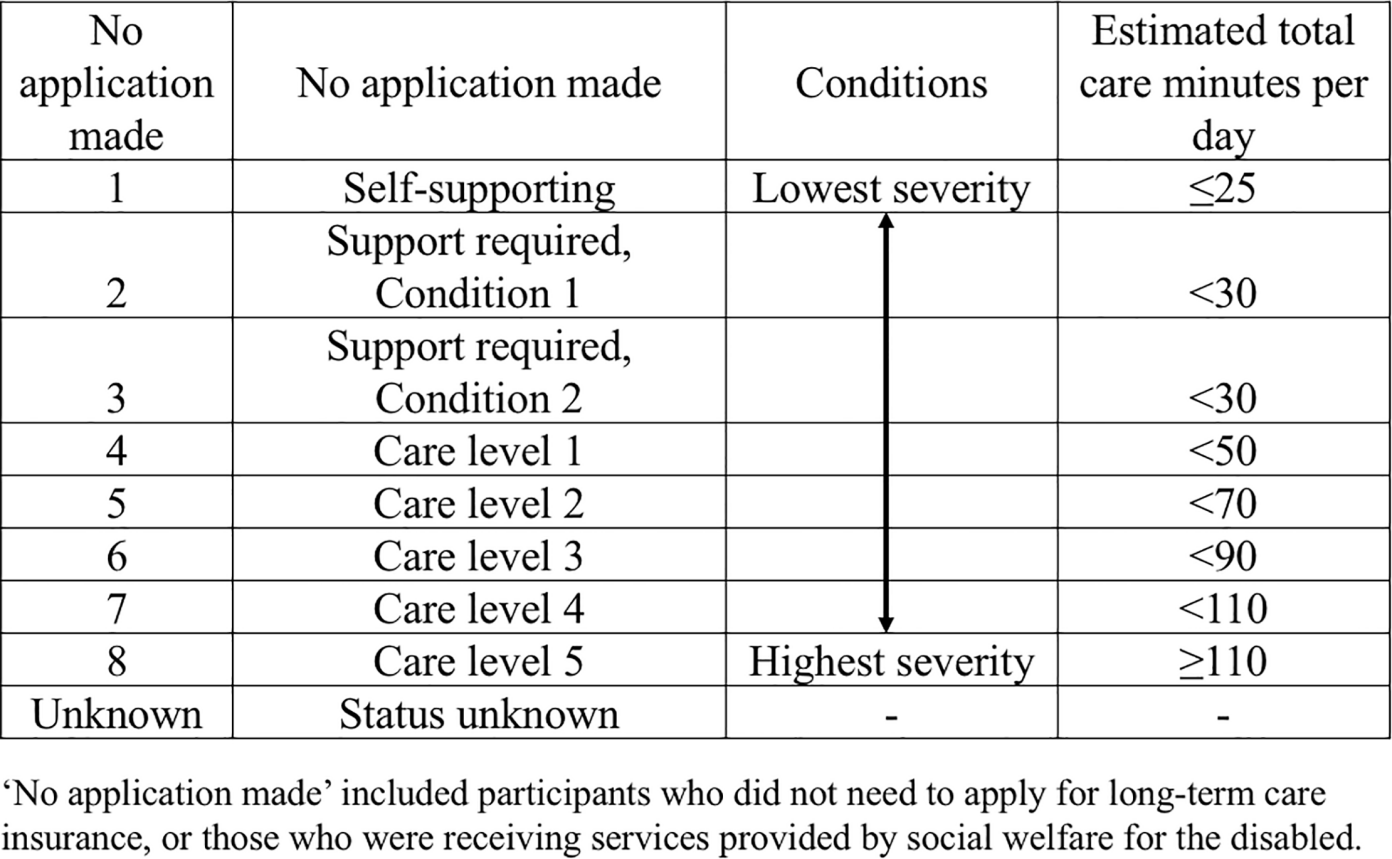
Need category for long-term care.

We conducted this cross-sectional survey in the city of Goto in the western part of Japan. Goto is situated on a remote, rural archipelago off the coast of Nagasaki prefecture. At the time of this study (2017), Goto had a population of 37,855 people, with a large proportion of people aged ≥65 years (37.7%). In 2000, the Japanese government initiated mandatory public Long-Term Care Insurance (LTCI) to help older people lead more independent lives and relieve the burden on family caregivers [19]. Since 1997, in keeping with LTCI policy, the Goto city municipal government has provided long-term care services for community-dwelling adults aged 65 years or older who have difficulty at home and are accredited as certified care recipients by a committee based on the LTCI Act [20]. Eligibility for LTCI certification is assessed with a 74-item questionnaire covering activities of daily living. Applicants undergo preliminary categorisation into eight levels by a computer algorithm, with this categorisation reviewed and finalised by a municipality-based expert committee [19]. This committee determines the ‘Category of Condition of Need for Long-Term Care’ (need category) for each LTCI application (**Fig 1**). The quantity of services covered for each person is based on their need category [20]. Need categories are defined in a range that includes ‘Self-supporting’, ‘Support required, Condition 1 or 2’ and ‘Care levels 1 through 5’ (low to high grading). Self-supporting indicates those at risk of being in need of support/long-term care [20, 21]. Support required Condition 1 or 2 represents those at risk of being in need of long-term care and requiring daily living support [20]. Care level 1 through 5 indicates those who are bedridden or people with dementia requiring long-term care services [20]. Each level sets a ceiling amount of services that can be purchased as benefits, ranging from US$400 to US$2900 per month [19].

In the present study, we approached four hospitals (of four), nine facilities covered by LTCI (of 10), three home-visit nursing services (of four), three group homes for older adults with dementia (of 23), and four geriatric facilities not covered by LTCI (of 25) in Goto. We included all residents aged ≥18 years from each facility. We excluded residents aged <18 years or those who were provided services from the home-visit nursing service station where permission for use of data could not be obtained.

### Definition of pressure injury prevalence

In this study, we defined prevalence as ‘point prevalence’, which indicates the proportion of a defined set of people who have pressure injuries at a particular moment in time [12]. This is expressed as a value per 1000 members of the population studied. The equation for point prevalence is: ‘point prevalence (%)’ = ‘number of persons with a pressure injury at the given time point’ / ‘total number of persons in the population studied at the same time point’ * 100. Because of auditing difficulties, we made an exception in the case of patients visited by home care nurses. ‘Period prevalence’ was used for these patients rather than point prevalence. Period prevalence indicates the number of patients who had pressure injuries at any time during a specified period (the previous 1 month at the time of the survey) [12].

### Defining the population as the denominator for prevalence

Definition of the population as the denominator for prevalence will have a strong impact on the results and conclusions. The presence of independent risk factors (e.g. age, mobility, diabetes, skin moisture, nutrition and general health status) in the study population should not be ignored [9]. Facility-based prevalence studies cannot avoid differences in composition of pre-existing risk factors in the defined populations. Therefore, defining the study population as all adults aged ≥18 years including medical or long-term care service users and healthy people will be less biased, and provide methodologically robust prevalence data. Given the demographic change in Japan, we also calculated the prevalence of pressure injuries in older people aged ≥65 years, which will aid in comparing prevalence as a benchmark value with other areas or countries.

### Data collection and identification of pressure injuries

We collected data prospectively from 21 August to 28 September 2017. All eligible participants were assessed on the date of visit by the research team (SN, HY, KS) using a structured investigation sheet. All grading was performed by at least two researchers, with KS always included in the surveyors. We visited facilities and directly observed participants at the time of regular physical examination by attending doctors or during routine care by nurses. However, for the home-visit nursing stations, it was difficult to visit all eligible patients’ homes; therefore, the researchers evaluated summarised medical charts. We could obtain precise data using the DESIGN-R grading system in these cases because Japanese home-visit nursing includes mandatory assessment of possible pressure injuries using evaluation forms. Data on age, sex, date of admission, LTCI need category and presence or absence of pressure injuries were recorded. To avoid double counting of cases that were admitted or discharged to/from acute care hospitals, we searched the history of transitions for the previous 1 month. In participants with pressure injuries, we collected data on the location of the pressure injuries and classification using the DESIGN-R schema, which includes attributes such as depth, exudate, size, inflammation, granulation tissues, necrotic tissue and pockets [22]. DESIGN-R is a seven-item (depth, exudates, size, inflammation/infection, granulation, necrosis and pocket) monitoring scale for pressure injuries developed by the Scientific Education Committee of the Japanese Society of Pressure Ulcers [22]. It can be used to score the severity of pressure injuries and monitor the healing process. Six of the DESIGN components (excluding depth) are weighted according to their relationship to the healing rate, and these scores can be summed to create a total DESIGN-R score, ranging from 0 (healed) to 66 (greatest severity) [22]. To detect non-blanchable erythema, we used a transparent interface to apply light pressure. Incontinence-associated dermatitis and medical device-related pressure injuries were not counted as pressure injuries. To calculate the prevalence of pressure injuries, we counted each individual as one case, even if that person had multiple pressure injuries.

### Estimation procedure for pressure injury prevalence among community-dwelling adults in Goto

**Fig 2:**
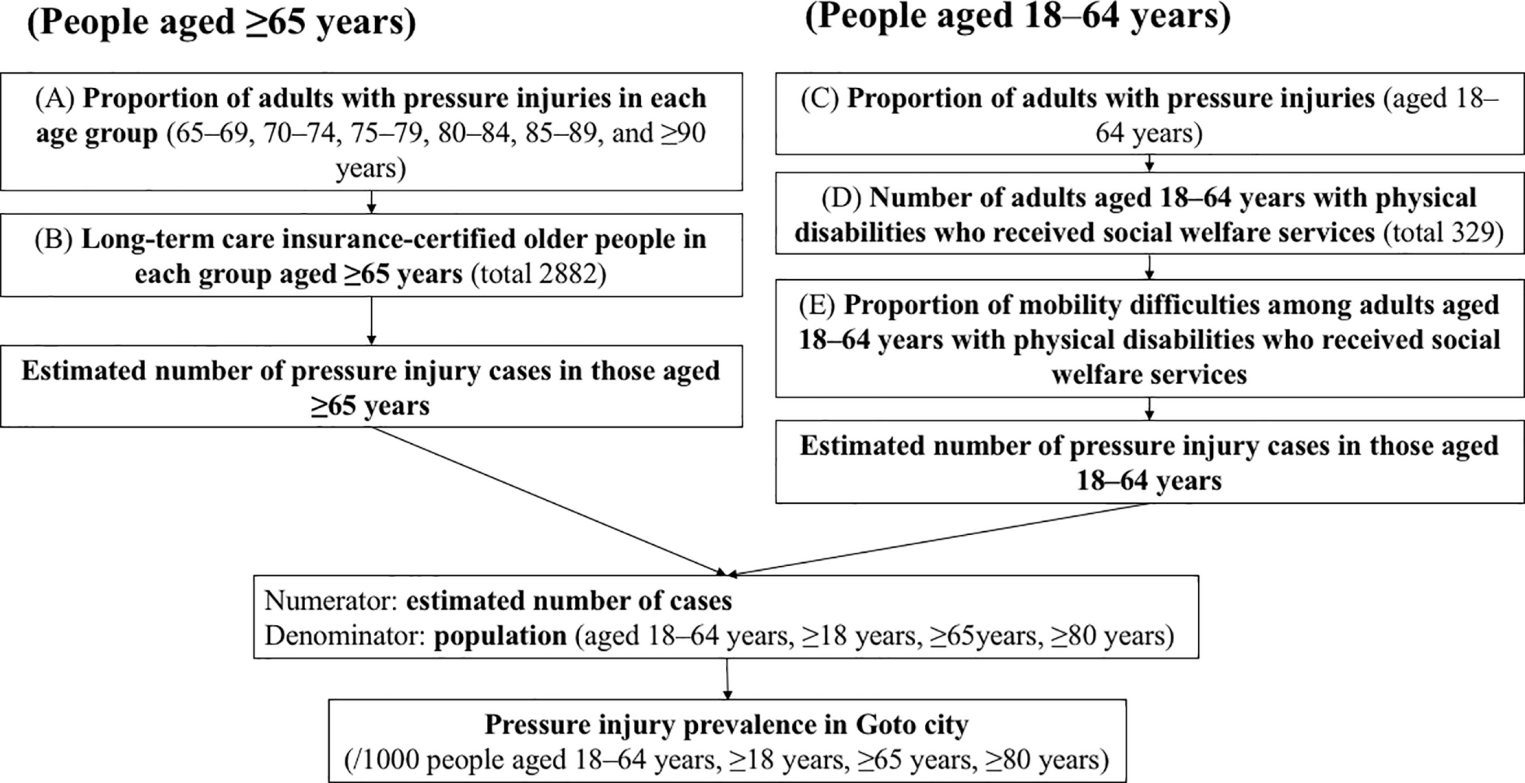
Procedure used to estimate the prevalence of pressure injuries in Goto.

Pressure injury prevalence was estimated using the calculation method described below. First, we calculated the proportion of adults aged ≥65 years with pressure injuries in six age groups (65–69, 70–74, 75–79, 80–84, 85–89 and ≥90 years) using data for 1126 cases (A in **Fig 2**). To estimate the number of cases with pressure injuries in Goto, we assumed all cases aged ≥65 years were LTCI-certified older people. The number of LTCI-certified older people in each age group (total 2882) were then used for further calculation (B in Fig 2). We calculated the estimated number of cases in Goto by multiplying A and B in each age category.

Next, we calculated the proportion of adults aged 18–64 years with pressure injuries using data for 1126 cases (C in Fig 2). To estimate the number of cases with pressure injuries in Goto, we assumed all cases aged 18–64 years were adults with physical disabilities who received social welfare services. The number of adults aged 18–64 years with physical disabilities who received social welfare services (D in Fig 2) and the proportion of those with mobility difficulties (E in Fig 2) was obtained from a postal survey conducted by the Goto city government between 25 July 2016 and 26 August 2016 [23]. We calculated the estimated number of cases in Goto by multiplying C, D and E.

To obtain the estimated prevalence of pressure injuries in adults aged ≥18 years, the numerator (estimated number of cases) was divided by the denominator (population in the city aged ≥18 years) (32,926). The estimated prevalence rates of pressure injuries in adults aged 18–64 years, adults aged ≥65 years and adults aged ≥80 years were calculated based on the population aged 18–64 years (18,673), ≥65 years (14,768) and ≥80 years (5,985), respectively.

The population was defined according to the Goto City Census (reported on 1 August 2017). The number of LTCI-certified older people was defined according to the Goto City Report on LTCI (31 July 2017). The 95% confidence intervals (CI) for the prevalence rates were calculated using Poisson regression. All P-values for statistical tests were two-tailed, and P < 0.05 was considered statistically significant. All statistical analyses were performed using STATA v14 (StataCorp, College Station, TX, USA).

### Ethics

This study was approved by the Institutional Review Board of Nagasaki University, Nagasaki, Japan (project registration number: 14051404). The requirement for obtaining written consent from patients was waived by the Institutional Review Board because of the study’s observational nature and lack of deviation from current medical practice or usual care. We used anonymised data for the analyses.

## Results

We visited 980 residents in the participating facilities and checked 149 home-visit nursing records. Three cases were excluded because they were aged <18 years. **Table 1** shows the characteristics of participants evaluated by the research team. In total, 1126 participants (median age 85 years, 31.4% male) were assessed, which accounted for more than one-third of LTCI-certified older people (n = 2882) in Goto. Half of the participants resided in geriatric facilities, 30% in hospital, and 13% at home. Around 85% of participants had applied for LTCI. The two most severe need categories for LTCI services (patients with mobilisation difficulties) accounted for 40% of all participants. Of these, 10% (n = 113) had one or more pressure injuries.

**Table 1:** Characteristics of participants evaluated by the research team (n = 1126).

Variables	n (%)
Age (years)	
Median (25th percentile, 75th percentile)	85 (76, 90)
Minimum, maximum age	25, 105
Men	354 (31.4)
Residential facility*	
Acute care hospitals	177 (15.7)
Acute/sub-acute care hospitals	151 (13.4)
Geriatric facilities LTCI+	525 (46.6)
Home	148 (13.1)
Geriatric facilities LTCI−	71 (6.3)
Group homes	54 (4.8)
Need category for long-term care**	
No application made	134 (11.9)
Self-supporting	84 (7.5)
Support required, Condition 1	28 (2.5)
Support required, Condition 2	28 (2.5)
Care level 1	120 (10.7)
Care level 2	116 (10.3)
Care level 3	155 (13.8)
Care level 4	223 (19.8)
Care level 5	203 (18.0)
Unknown	35 (3.1)
Pressure injuries	113 (10.0)

Data are presented as n (%); LTCI, Long-Term Care Insurance.

*Acute care hospitals were defined as bed capacity ≥100; acute/sub-acute care hospitals, as bed capacity <100; geriatric facilities LTCI+, long-term care geriatric facilities covered by LTCI; home, home care supported by home-visit nursing services; geriatric facilities LTCI−, long-term care geriatric facilities not covered by LTCI; group homes, group homes for older adults with dementia.

** ‘No application made’ includes participants who did not need to apply for LTCI, or those who were receiving services provided by social welfare for the disabled. Need categories for long-term care are classified into ‘self-supporting’ (lowest severity) to ‘care level5’ (highest severity). They are defined by estimated total care minutes per day (≤25, <30, <30, <50, <70, <90, <110 and ≥110 minutes).

Details of identified pressure injuries are summarised in **Table 2**. Of 113 identified pressure injuries, the most frequently reported severity level was ‘persistent redness’ (42%), followed by ‘lesion extends to dermis’ (31%). In 60% of cases, the injury size was smaller than 4 cm^2^, and there were only four cases with a pocket. The median total DESIGN-R score was 6 (**Table 3**). The dominant sites for pressure injuries were the sacrum, lower leg/ankle, hips, vertebral region and foot. **Table 4** shows the facility-based proportion of pressure injuries. Geriatric facilities not covered by LTCI, acute/sub-acute care hospitals, and geriatric facilities covered by LTCI had higher proportions of people with pressure injuries than acute care hospitals (14.1%, 13.9%, 12.2% and 6.2%, respectively).

**Table 2:** DESIGN-R classification of participants’ pressure injuries (n = 113).

DESIGN-R classification	n	%
Depth		
d0	0	0
d1	47	41.6
d2	35	31.0
D3	18	15.9
D4	5	4.4
D5	0	0
DU	8	7.1
Exudate		
e0	77	68.1
e1	30	26.6
e3	5	4.4
E6	1	0.9
Size*		
s0	1	0.9
s3	66	59.5
s6	33	29.7
s8	6	5.4
s9	4	3.6
s12	0	0
S15	1	0.9
Inflammation or infection		
i0	108	95.6
i1	4	3.5
I3	1	0.9
I9	0	0
Granulation tissue**		
g0	84	74.3
g1	5	4.4
g3	3	2.7
G4	2	1.8
G5	3	2.7
G6	16	14.2
Necrotic tissue*,***		
n0	84	75.0
N3	21	18.8
N6	7	6.3
Pocket*,****		
p0	108	96.4
P6	2	1.8
P9	2	1.8
P12	0	0
P24	0	0

* Data were missing for size of pressure injury (n = 2), necrotic tissue (n = 1) and pockets (n = 1).

**Granulation tissue: percentage of healthy granulation.

***Necrotic tissue: when necrotic and non-necrotic tissues were mixed, the dominant condition was used for assessment.

****Pocket: area obtained by subtracting the injury from the entire affected area, including the pocket.

d0 indicates no particular skin legion and no redness; d1, persistent redness; d2, lesion extends to dermis; D3, lesion extends into subcutaneous tissue; D4, lesion extends to muscle, tendon and bone; D5, lesion extends into the articular or body cavity; DU, impossible to measure depth; e0, none; e1, slight, does not require daily dressing change; e3, moderate, requires daily dressing change; E6, heavy: requires dressing change more than twice/day; s0, none; s3, smaller than 4cm^2^; s6, 4 cm^2^ or larger, but smaller than 16cm^2^; s8, 16 cm^2^ or larger, but smaller than 36cm^2^; s9, 36 cm^2^ or larger, but smaller than 64cm^2^; s12, 64 cm^2^ or larger, but smaller than 100cm^2^; S15, 100 cm^2^ or larger; i0, none; i1, signs of inflammation (fever, redness, swelling, pain around the wound); I3, clear signs of local infection (inflammation, pus, foul smell); I9, systemic impact, such as fever; g0, granulation cannot be assessed because the wound was healed or too shallow; g1, healthy granulation tissue occupies 90% or more; g3, healthy granulation tissue occupies 50% or more, but less than 90%; G4, healthy granulation tissue occupies 10% or more, but less than 50%; G5, healthy granulation tissue occupies less than 10%; G6, no healthy granulation tissue exists; n0, none; N3, soft necrotic tissue exists; N6, hard and thick necrotic tissue attached to the wound; p0, none; P6, smaller than 4 cm^2^; P9: 4 cm^2^ or larger, but smaller than 16 cm^2^; P12,16 cm^2^ or larger, but smaller than 36 cm^2^; P24, 36 cm^2^ or larger.

**Table 3:** Total DESIGN-R score and body location of pressure injuries (n = 113).

Total DESIGN-R score*		
Median (25th percentile, 75th percentile)		6 (3, 9)
Minimum, maximum score		0, 32
Body location of pressure injuries	N	%
Sacrum	57	50.4
Lower leg/ankle	21	18.6
Hips	13	11.5
Vertebral region	9	8.0
Foot	5	4.4
Hand	3	2.7
Buttock	3	2.7
Elbow	1	0.9
Knee	1	0.9

*Data on DESIGN-R score were missing in two cases.

**Table 4:** Proportion of people with pressure injuries by age group, LTCI need category and residential facility (n = 1126).

Variables	Total	Cases	%
Age (years)			
18–64	138	12	8.7
65–69	45	2	4.4
70–74	64	2	3.1
75–79	113	8	7.1
80–84	193	22	11.4
85–89	254	29	11.4
≥90	319	38	11.9
Need category for long-term care*			
No application made	134	4	3.0
Self-supporting	84	2	2.4
Support required, Condition 1	28	1	3.6
Support required, Condition 2	28	1	3.6
Care level 1	120	5	4.2
Care level 2	116	16	13.8
Care level 3	155	13	8.4
Care level 4	223	27	12.1
Care level 5	203	41	20.2
Unknown	35	3	8.6
Residential facility**			
Acute care hospitals	177	11	6.2
Acute/sub-acute care hospitals	151	21	13.9
Geriatric facilities LTCI+	525	64	12.2
Home	148	3	2.0
Geriatric facilities LTCI−	71	10	14.1
Group homes	54	4	7.4

LTCI, long-term care insurance.

* ‘No application made’ included participants did not need to apply for LTCI, or those who were receiving services provided by social welfare for the disabled. Need categories for long-term care are classified into ‘self-supporting’ (lowest severity) to ‘care level5’ (highest severity). They are defined by estimated total care minutes per day (≤25, <30, <30, <50, <70, <90, <110 and ≥110 minutes).

**Acute care hospitals defined as bed capacity ≥100; acute/sub-acute care hospitals, as bed capacity <100; geriatric facilities LTCI+, long-term care geriatric facilities covered by LTCI; home, home care supported by home-visit nursing services; geriatric facilities LTCI−, long-term care geriatric facilities not covered by LTCI; group homes, group homes for older adults with dementia.

The age-stratified proportion of people with pressure injuries was based on evaluation of 1126 participants by the researchers (A and C in Fig 2, Table 4). The oldest age group had the highest proportion of people with observed pressure injuries (11.9% in those aged ≥90 years). The estimated number of pressure injury cases among those aged ≥65 years was 299.7, which was the sum of the values derived by multiplying the proportion of adults aged ≥65 years with pressure injuries and LTCI-certified older people in the city in each age group (A and B in Fig 2, **Table 5**). The estimated number of pressure injury cases in those aged 18–64 years was 1.7. This was calculated by multiplying the proportion of pressure injuries in those aged 18–64 years (0.087), the number of adults with physical disabilities aged 18–64 years who received social welfare services (329) and the proportion of adults aged 18–64 years with physical disabilities and mobility difficulties who received social welfare services (0.06) (C, D and E in Fig 2, Table 5).

**Table 5:** Estimated pressure injury prevalence rates per 1000 population in Goto, by age group.

Age group (years)	Susceptible population*	Proportion of pressure injuries	Estimated number of cases	Estimated prevalence rate
18–64	20	8.70	1.7	
65–69	106	4.44	4.7	
70–74	142	3.12	4.4	
75–79	329	7.08	23.3	
80–84	629	11.4	71.7	
85–89	833	11.42	95.1	
≥90	843	11.91	100.4	
Total			301.4	
Adults aged ≥ years18 in Goto	32 926			9.2 (95% CI 8.1–10.2)
Older people aged ≥65 years in Goto	14 768			20.3 (95% CI 18.1–22.7)
Old-old people aged ≥80 years in Goto	5985			44.6 (95% CI 39.5–50.2)

CI, confidence interval.

*Susceptible population for the age group 18–64 years was derived by the number of people with physical disabilities who were receiving services provided by social welfare. Susceptible populations for the other age categories were the number of Long-Term Care Insurance-certified older people.

Total population of Goto is based on Goto city statistics as of 1 August 2017.

Finally, we divided the numerator (estimated number of cases) by the denominator (populations in the city aged ≥18, ≥65, ≥80, or 18–64 years) to obtain the estimated pressure injury prevalence among adults in Goto. The prevalence of pressure injuries was 9.2 per 1000 population in adults aged ≥18 years (95% CI 8.1–10.2), 20.3 in older people aged ≥65 years (95% CI 18.1–22.7), 44.6 in old-old people aged ≥80 years (95% CI 39.5–50.2) and 0.1 in adults aged 18–64 years (95% CI 0.0–0.4).

## Discussion

The present study is the first to evaluate the population-based prevalence of pressure injuries in Japanese adults and older adults. Rapid demographic change from population aging is a major factor driving the disease burden of pressure injuries as an age-related disease in Japan. In this context, population-based prevalence data will be useful in planning resource allocation and countermeasures for pressure injuries.

Compared with a previous population-based study in the United Kingdom, our results revealed a high prevalence of pressure injuries in this rural Japanese community. The previous UK cross-sectional study showed a pressure injury prevalence of 0.77 per 1000 adults aged ≥18 years in an urban site (population 292,179), and 0.40 per 1000 adults in a rural site (population 311,991) [24]. These rates were lower than those in our study. Possible reasons for this may include differences in: 1) case identification methods; 2) types of selected facilities; and 3) susceptible populations in the study areas. With regard to case identification method, the UK study depended on community nursing caseloads, whereas our study was mainly based on direct observation. The proportion of injuries showing ‘persistent redness’ or ‘lesion extending to the dermis’ was 70.6% (40.6% and 30.0%, respectively) in our study, whereas it was 75.4% (35.2% and 40.2%, respectively) in the UK study. This suggests that missing cases of mild pressure injuries are less likely with an indirect method of case identification as used in the UK study. In terms of selected facilities, we approached hospitals, geriatric facilities covered by LTCI, home-visit nursing services and group homes for older adults with dementia. In contrast, the UK study approached six healthcare providers in the community (community nursing services, residential homes, rehabilitation units, specialist palliative care units, nursing homes and general practitioners). The inclusion of acute care patients in the hospital population might explain a considerable part of the difference between our study and the UK study. Excluding participants recruited in hospitals, we estimated a prevalence of 9.1 (95% CI 4.1–17.0) pressure injuries per 1000 adults using an age-specific calculation method. Regarding susceptible populations, an important factor is population aging. In total, 37.7% of the population in Goto was aged ≥65 years. In the UK study, the population aged ≥65 years was 14.2% in the urban area and 20.2% in the rural area. When the prevalence of pressure injuries was only calculated among those aged ≥65 years in the UK study, the prevalence was 3.7 per 1000 population in older people aged ≥65 years old at the urban site and 2.1 per 1000 population at the rural site. These findings highlight that caution is needed to report comparable epidemiological data on pressure injury prevalence along with the precise age structure of the susceptible population. Although the sample sizes in both the UK study and our study were large, estimation of age-adjusted prevalence in our study was not feasible because of lower reliability in some age groups with small numbers of cases. We estimated the prevalence rate among older people aged ≥65 years as a feasible alternative method.

To date, published facility-based prevalence data for pressure injuries based on a methodologically reliable design in Japan are limited, and there is a diverse range of reported prevalence rates [16–18]. The Japanese Society of Pressure Ulcers investigated the facility-based prevalence of pressure injuries in 397 facilities in Japan. That study found variation in facility-based prevalence among types of facilities (from 1.89% to 5.45%) [17]. In another study, the period prevalence in long-term care hospitals in Japan was reported as 9.6% per month [18]. A retrospective hospital-based prevalence study in Japan showed the prevalence decreased from 4.26% to 3.64% 1 year after the introduction of a penalty system for reported pressure injuries by the Japan Ministry of Health, Labour and Welfare. This result supports the idea that the prevalence of pressure injuries can be influenced by governmental policy.

Another important finding in this study was that the proportions of people with pressure injuries in geriatric care facilities or long-term care hospitals were higher than that in acute care hospitals. The higher prevalence in long-term care facilities may be explained by: 1) limited resources and insufficient systematic prevention strategies in long-term care facilities in Japan; and 2) frequent care transitions from hospitals to long-term care facilities.

Recently, Japan’s Ministry of Health, Labour and Welfare made major systematic advances in the prevention of and treatment for pressure injuries including: incentives and penalties for systematic assessment to prevent pressure injuries and standardised care for all inpatients [25]; a common medical format to record assessment and treatment plans; monthly audits by hospital committees on pressure injuries; use of affordable alternating air mattresses; frequent educational sessions; and increasing the number of nurses certified for wound, ostomy and incontinence care. These strategies succeeded in reducing the prevalence of pressure injuries and were cost-effective [16, 26].

Long-term care facilities and long-term care hospitals have fewer nursing staff than acute care hospitals. A cross-sectional study showed that 24-h nursing care was only provided in 17.9% of long-term care facilities [27]. In long-term care hospitals, 35% of the interdisciplinary teams were not useful, more than half of the clinical protocols were not used frequently and about half of the wards did not have enough pressure-relieving mattresses [18]. In addition, care transitions from hospitals to long-term care facilities are often required for older people [14]. In a facility-based period prevalence study in Japan, 20.8% of pressure injuries in hospitals were detected at the time of admission [17]. The fact that wound healing required an average of over 3 weeks after admission to hospitals in Japan [26] suggests that most patients admitted to acute care hospitals in Japan do not complete their healing process while hospitalised. This means that most pressure injuries emerge in long-term care facilities or long-term care hospitals, and consequently need to be treated there. Preventive strategies to reduce the disease burden in the community should focus on long-term care facilities and long-term care hospitals.

### Strengths and limitations

An important strength of this study was the population-based data collection method. Participating study sites included all four hospitals in Goto, three of the four home care services and nine of the 10 geriatric health service facilities (those with a ≥30 bed capacity). Unfortunately, the limited number of research staff did not allow us to evaluate all health service facilities (such as group homes for older people with dementia with a bed capacity <10; we investigated three of 23). Second, the prospective data collection design of the present study was more accurate than retrospective data collection. Retrospective data collection may produce underestimates because a significant proportion of pressure injuries are not documented [28]. Third, the prospective data collection also assured diagnostic accuracy. Secondary use of existing data is less accurate because: 1) non-blanchable erythema identified by clinical staff may be underestimated because of lack of awareness of the signs, 2) fear of personal or institutional recrimination may cause under-reporting of cases; and 3) there is possibility of misclassification as other dermatological differential diagnoses [12]. Fourth, the rating system in our study might have helped to improve inter-rater reliability. All participants were assessed by three of the present researchers (SN, HY, KS) to control the quality of inter-rater reliability. We also visited all facilities in pairs, one of which was KS, who is a certified nurse in wound, ostomy and incontinence care. For cases that were initially evaluated by SN or HY, ratings were subsequently discussed with KS before being finalised and recorded. Therefore, the data were expected to have higher inter-rater reliability. Finally, overestimation of cases by case-mix (double counting) did not bias our results because the survey was conducted over 6 weeks and we monitored all transitions of participants among facilities.

The main limitation of this study was its estimation method, which carries a theoretical risk of underestimating the proportion of cases. In estimating prevalence, we assumed that all pressure injury cases were those under the certification of LTCI or those who were physically disabled and receiving social welfare services. This means that pressure injuries in people that were not certified by LTCI or who did not receive social welfare services were not counted.

## Conclusion

This study revealed a high level of population-based prevalence of pressure injuries in a rural Japanese community. A methodologically rigorous design using prospective direct observation by a research team in a range of facilities supported reliable estimation. We propose that the background population who are susceptible to pressure injuries should also be considered. An age-specific report (using age adjustment or stratification of those aged <65 years and those aged ≥65 years) will be helpful in future comparisons.
